# Self-assembled nanorods in YBCO matrix – a computational study of their effects on critical current anisotropy

**DOI:** 10.1038/s41598-020-59879-3

**Published:** 2020-02-21

**Authors:** Elmeri Rivasto, Mukarram Zaman Khan, Mika Malmivirta, Hannes Rijckaert, Moe Moe Aye, Teemu Hynninen, Hannu Huhtinen, Isabel Van Driessche, Petriina Paturi

**Affiliations:** 1 Wihuri Physical Laboratory, Department of Physics and Astronomy, University of Turku, FI-20014 Turku, Finland; 2 University of Turku Graduate School (UTUGS), University of Turku, FI-20014 Turku, Finland; 3 SCRiPTS, Department of Chemistry, Ghent University, Krijgslaan 281 S3, 9000 Ghent, Belgium

**Keywords:** Superconducting properties and materials, Electronic properties and materials, Computational methods, Surfaces, interfaces and thin films

## Abstract

In order to understand how the doping with self-assembled nanorods of different sizes and concentrations as well as applied magnetic fields affect the critical current anisotropy in YBa_2_Cu_3_O_7−*x*_ (YBCO) thin films close to YBCO *c*-axis, we present an extensive and systematic computational study done by molecular dynamics simulation. The simulations are also used to understand experimentally measured *J*_*c*_(*θ*) curves for BaHfO_3_, BaZrO_3_ and BaSnO_3_ doped YBCO thin films with the help of nanorod parameters obtained from transmission electron microscopy measurements. Our simulations reveal that the relation between applied and matching field plays a crucial role in the formation of *J*_*c*_(*θ*)-peak around YBCO *c*-axis (*c*-peak) due to vortex-vortex interactions. We also find how different concentrations of different size nanorods effect the shape of the *c*-peak and explain how different features, such as double *c*-peak structures, arise. In addition to this, we have quantitatively explained that, even in an ideal superconductor, the overdoping of nanorods results in decrease of the critical current. Our results can be widely used to understand and predict the critical current anisotropy of YBCO thin films to improve and develop new pinscapes for various transport applications.

## Introduction

High temperature superconductors (HTS) are expected to have large number of applications in different fields of technology and power industry in the future^1–3^. Since all known HTS are of type II, the critical current passed through them is highly dependent on the surrounding magnetic field due to the movement of vortices. Thus, to enhance and widen the usability of HTS, the dynamics of vortices need to be well understood.

Among the high temperature superconductors, YBa_2_Cu_3_O_7−*x*_ (YBCO) seems the most practical choice when thinking for the applications^1^. The intrinsic anisotropy of the critical current, in thin films and coated conductors, can be modified by adding impurities within the lattice of YBCO which pin the vortices restricting their movement. Based on growth conditions and lattice mismatch between the YBCO and the dopant as well as their elastic properties^4,5^, impurities such as Y_2_O_3_^6^, BaCeO_3_^7–9^ and BaZrO_3_ (BZO)^10,11^ can form uncorrelated randomly distributed nanoparticles within the YBCO lattice. Under optimized deposition conditions, via a spontaneous phase-separation and strain-driven self-assembly process during film deposition^12^, self-assembly of nanorods of BaHfO_3_ (BHO)^1^, BaZrO_3_ (BZO)^4,13,14^, BaSnO_3_ (BSO)^15,16^, Ba_2_YTaO_3_ (BYTO)^17^ or Ba_2_YNbO_6_ (BYNO)^18^ within the YBCO lattice can be realized.

Recently, a topic of interest has been to add both point-like nanodots and nanorods within the YBCO lattice simultaneously. This has been achieved by doping YBCO simultaneously with both BYTO and BYNO (referred as BYNTO) with an additional rare earth oxide, leading to continuous niobiate/tantalate nanorods and rare-earth oxide nanoparticles^19^. A lot of experimental research has been done in order to understand the mechanisms of flux pinning related to these materials. A huge topic of interest has been how different dopants affect the isotropy of angular dependent critical current *J*_*c*_(*θ*).

While the effect of different pinning centers on the dynamics of the vortices in YBCO thin films is theoretically rather well known, their effects on *J*_*c*_(*θ*) are not comprehensively understood. *J*_*c*_(*θ*) has been modeled before by the statistical vortex path model^20–22^, which can be used to explain the shapes of *J*_*c*_(*θ*) curves but it does not provide any information about the dynamics of the vortices. The field dependency of critical currents in BZO doped YBCO lattice has also been computationally investigated by numerically solving the Ginzburg-Landau equations in^23^, but the effect of the applied field on critical current anisotropy has not been discussed. In the paper^25^, where the validation of the molecular dynamics (MD) simulation used in this work is presented, the effect of nanorod diameter was shortly studied as a demonstration of the feasibility of the simulation, but a detailed analysis on this subject is still lacking. The simulation model has also been used in our previous work^9^ to analyse critical current anisotropies in multilayer films containing both nanodots and nanorods and the effect of the splay and fragmentation of the nanorods on *J*_*c*_(*θ*)-peak in the vicinity of YBCO *c*-axis (*c*-peak) has been studied in^24^.

In this article, we present the first extensive and systematic study of the effects of the applied magnetic field, nanorod diameter and concentration on the shape of YBCO *c*-peak, also providing detailed knowledge about the actual dynamics of the vortices. Our results can be used to explain features of experimentally measured *J*_*c*_(*θ*) curves or vice versa to analyze the microstructure of the nanorods in HTS thin films. In addition, we demonstrate the quality of our model by simulating the *J*_*c*_(*θ*) curves of BHO, BZO and BSO doped YBCO films using sizes and distributions of the nanorods obtained from BF-STEM images. Finally, we present a direct comparison of simulated critical current anisotropies to the experimentally measured ones.

## Results and Discussion

### Effects of nanorods

One of the most common features of nanorods experimentally encountered in thin films is their splay and fragmentation, the effects of which to YBCO *c*-axis peak have already been comprehensively studied in^24^, where the increasing splay of nanorods was reported to decrease the absolute value of critical current. Double peak structures were observed for all tilting angles of nanorods, but interestingly this double peak structure was most pronounced without any splay at zero angle. The fragmentation of the nanorods was reported to destroy the double peak structure and decrease the anisotropy of the *J*_*c*_(*θ*) curve without much effect to the absolute value of critical current.

**Figure 1:**
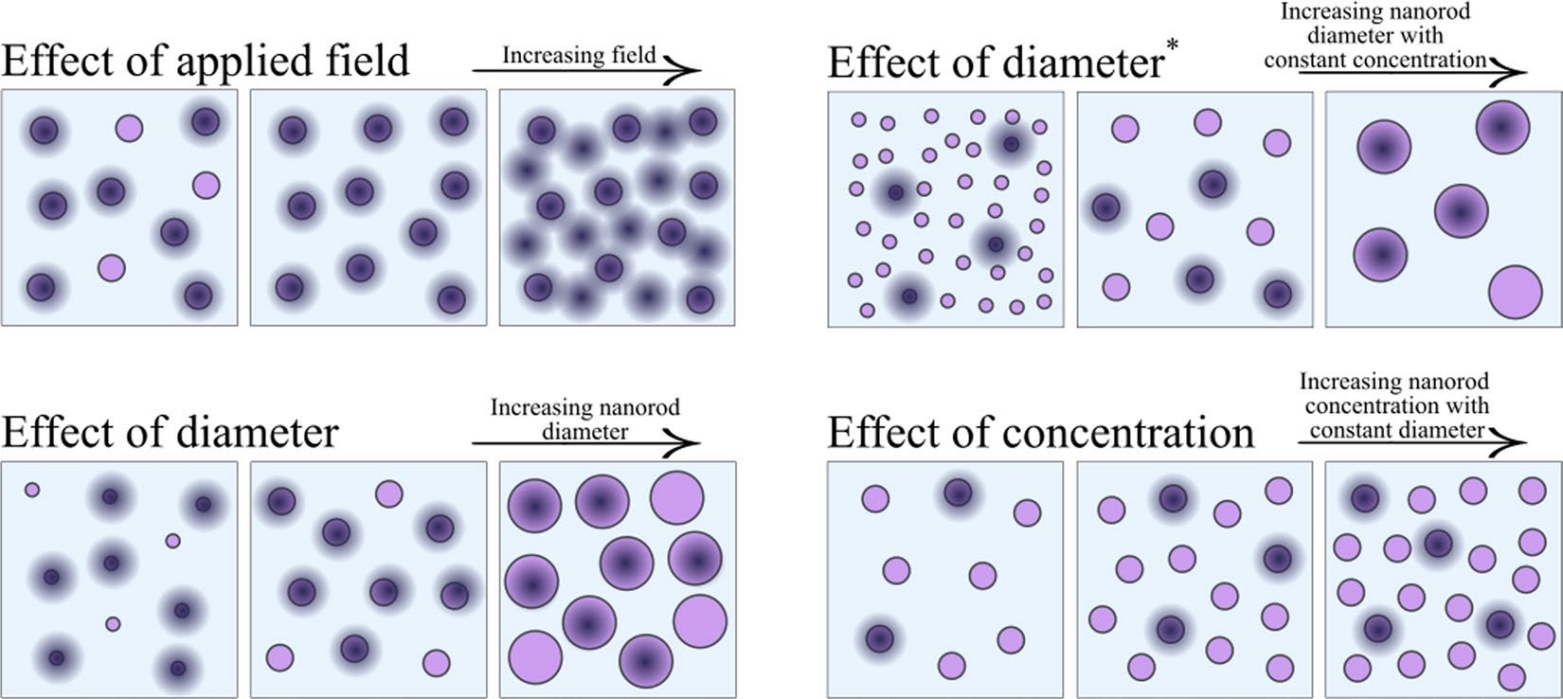
The schematic diagram of simulations run in the subsections below. Purple dots represent a plain view of nanorods and black shady dots vortices.

The effect of nanorod diameter on YBCO *c*-peak has also been studied before in^25^, but unfortunately this work cannot be used to draw any conclusions if the observed effects are due to nanorod diameter or their varying amount, which is a function of nanorod diameter if concentration is kept constant. Thus, in this work we firstly study the raw effect of nanorod diameter by five different simulations that had constant number of nanorods but with varying sizes in constant applied field, respectively. Secondly, the more realistic effect of nanorod diameter was studied by changing the number of different size nanorods so that their concentration would correspond to 4% and simulating the *J*_*c*_(*θ*) curve again in a constant applied field. The relative numbers of nanorods and vortices in these simulations are illustrated in Fig. 1.

Another feature that has been experimentally proven to have significant effect on the absolute value and shape of *J*_*c*_(*θ*) curve is the applied magnetic field^26^, to which the number of vortices that penetrate type-II superconductor is directly proportional. These vortices minimize their energy by pinning into nanorods introduced within the superconducting lattice. Since the formation of double flux quantum qiant vortices can be assumed to be energetically unfavorable, a single nanorod can occupy only one vortex at a time. Intuitively, the probability of a vortex getting pinned, which itself is proportional to critical current, must be proportional to vortex-free nanorods. After all the nanorods are occupied, the vortices can only pin weakly in the vortex lattice via vortex-vortex interaction. This intuitively implies that the magnitude of the applied magnetic field indeed has a huge impact on both the absolute value and anisotropy of the critical current. In order to study the effect of applied field to the critical current anisotropy, simulations with a varying number of vortices were run using lattices of 4 nm, 8 nm and 12 nm diameter nanorods oriented along YBCO *c*-axis.

While the effect of nanorod size has been previously studied in pinscapes of both constant and varying number of nanorods, the effect of concentration of certain diameter nanorods to the shape of the *c*-peak is anything but trivial. The effect of concentration on different size nanorods was studied by running simulations with three different nanorod sizes and concentrations. We varied the nanorod diameter between 4 nm–12 nm which corresponds to the range of values theoretically predicted and experimentally measured for BHO, BZO and BSO^5,27^ and concentration between 2% and 6% which have experimentally produced the most favourable results^26^. These simulations were run in different magnetic fields, so that the ratio between the number of nanorods and vortices were kept constant at 0.2. By doing this, we can eliminate the previously mentioned effect of the applied field and thus the pure effect of nanorod concentration can be studied. Results obtained from corresponding simulations are presented and analyzed in the subsections below.

The simulation parameters, namely the nanorod diameter *d* and the number of nanorods *N*_r_ and vortices *N*_v_, for different simulations A-I are presented in Table 1 and all the different simulations are schematically illustrated in Fig. 1.

**Table 1:** Nanorod diameters *d*, concentrations *c*, corresponding applied fields *B*, number of nanorods *N*_r_ and number of vortices *N*_v_ used in a specific simulations A–I. The units of nanorod diameter correspond to nanometers. (^*^) refers to varying amount of different size nanorods with constant atomic concentration.

Effect of field	A	B	C	D	E	F	G	H	I	J
*d* (nm)	4	4	4	4	4	8	8	8	8	8
*c* (%)	1.3	1.3	1.3	1.3	1.3	2.6	2.6	2.6	2.6	2.6
*B* (T)	0.06	0.21	0.26	1.05	1.56	0.26	0.52	0.78	1.05	1.29
*N*_r_	40	40	40	40	40	20	20	20	20	20
*N*_v_	1	4	5	20	30	5	10	15	20	25
	K	L	M	N	O					
*d* (nm)	12	12	12	12	12					
*c* (%)	4.1	4.1	4.1	4.1	4.1					
*B* (T)	0.06	0.16	0.32	0.47	0.63					
*N*_r_	14	14	14	14	14					
*N*_v_	1	3	6	9	12					
Effect of diameter	A	B	C	D	E					
*d* (nm)	4	6	8	10	12					
*c* (%)	0.6	1.4	2.6	4.1	6.0					
*B* (T)	0.72	0.72	0.72	0.72	0.72					
*N*_r_	20	20	20	20	20					
*N*_v_	14	14	14	14	14					
Effect of diameter^*^	A	B	C	D	E					
*d* (nm)	4	6	8	10	12					
*c* (%)	4	4	4	4	4					
*B* (T)	0.72	0.72	0.72	0.72	0.72					
*N*_r_	122	54	31	20	14					
*N*_v_	14	14	14	14	14					
Effect of concentration	A	B	C	D	E	F	G	H	I	
*d* (nm)	4	4	4	8	8	8	12	12	12	
*c* (%)	2	2	2	4	4	4	6	6	6	
*B* (T)	0.62	1.24	1.86	0.16	0.31	0.47	0.05	0.16	0.21	
*N*_r_	62	122	180	16	31	45	7	14	20	
*N*_v_	12	24	36	3	6	9	1	3	4	

### Effect of applied magnetic field

**Figure 2:**
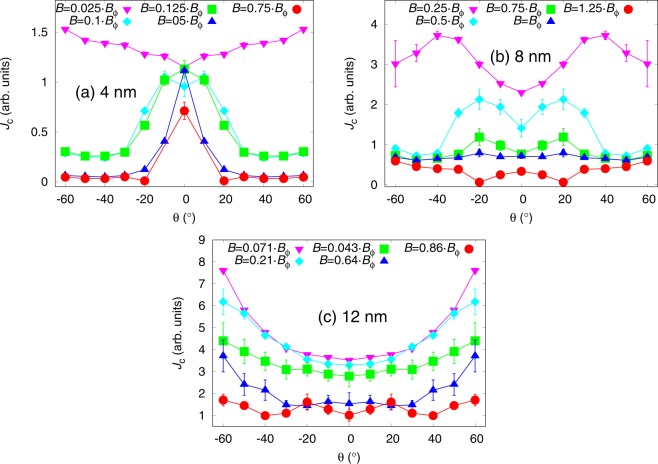
Simulated *J*_*c*_(*θ*) curves with standard errors for lattices of 4 nm, 8 nm and 12 nm diameter nanorods in different matching fields. Further details of the simulation conditions are presented in Table 1. The simulation failed at  ±10^°^ for 4 nm diameter nanorods at 0.75 ⋅ *B*_*ϕ*_ due to unstabilized vortex lattice, for which the data at that angle is not presented.

 Figure 2 presents the simulated *J*_*c*_(*θ*) curves for lattices of 40 nanorods with 4 nm diameter, 20 nanorods with 8 nm diameter and 14 nanorods of 12 nm diameter, all of which were perfectly solid and YBCO *c*-axis oriented, for varying magnetic fields. The previously mentioned lattices correspond to 1.3%, 2.6% and 4.1% doping concentrations, respectively. The simulations were run at various magnetic fields, but the effect of the relation of this field to the matching field *B*_*ϕ*_, that is the field in which the number of pinning center equals the number of vortices, was investigated. Further details of the individual simulations are listed in Table 1. The ratio between applied field and *B*_*ϕ*_ seems to have remarkable effect on the shape of the *J*_*c*_(*θ*) curve.

For 4 nm diameter nanorods, presented in Fig. 2a, the *c*-peak starts to form already in the low field range, around 0.1 ⋅ *B*_*ϕ*_. At field smaller than this, the *c*-peaks do not appear because of the weak pinning forces of the 4 nm wide nanorods that reduces the propability of a vortex getting pinned at low angles. As the angle is increased the propability of a vortex coming across a nanorod also increases thus increasing the *J*_*c*_ at high angles. Although the pinning forces of the 4 nm diameter nanorods are so low, that single vortex can not get pinned into several nanorods simultaneously, the increased propability of a vortex getting pinned in the first place is the reason behind the absence of the *c*-peak. At high fields, the *c*-peak arises due to repulsive vortex-vortex-interactions that, in the case of weak 4 nm diameter pinning sites, disturb the pinning of partly pinned vortices, that are present at high angles. One should also be pointed out that a single vortex was never witnessed to get pinned into more than one nanorod. Thus *c*-axis pinning is favoured. This also explains the sharpening of the *c*-peak as the field is increased.

In the case of 8 nm nanorods, as presented in Fig. 2b, the situation is more complex. At *B*_*ϕ*_, the *J*_*c*_(*θ*) curve is extremely isotropic and interestingly the *J*_*c*_(*θ*) curves seem to behave symmetrically with respect to *B*_*ϕ*_ as the applied field is increased or decreased. As the applied magnetic field is decreased from *B*_*ϕ*_, the absolute value of *J*_*c*_ increases at angles below 40^°^ giving rise to broadening double peak structure. The underlaying mechanism that gives rise to double *c*-peaks is not related to the magnitude of the applied field and is discussed comprehensively in the section 3. The broadening and increasing absolute values of the *c*-peaks, on the other hand, are the direct result of decreasing applied field. When *B* = *B*_*ϕ*_, the vortex lattice is so tight that vortices get very easily trapped also outside pinning centers due to vortex-vortex interaction. Since this interaction is somewhat independent of *θ*, the *J*_*c*_(*θ*) curve behaves very isotropically. When *B* < *B*_*ϕ*_, vortices cannot get trapped outside the pinning centers as effectively and pinning along the *c*-axis direction is preferred giving rise to more pronounced *c*-peaks. At very low fields, when *B* = 0.25*B*_*ϕ*_, the amount of vortices is so low that the interaction between them becomes negligible, allowing vortices to bend and get pinned into several nanorods simultaneously. This increases the pinning efficiency, and thus *J*_*c*_, especially at high angles. This implies that at very low fields the *J*_*c*_(*θ*) curve becomes more isotropic and the *c*-peak vanishes. When *B* > *B*_*ϕ*_, the simulation of *J*_*c*_(*θ*) curves becomes more difficult, especially at angles below 20^°^, due to the hard stabilization of the vortex lattice resulting in many of the simulations run at *B* = 1.25*B*_*ϕ*_ failing. Thus, because the lack of good statistics, the *J*_*c*_(*θ*) curve for this specific field in Fig. 2 should be adopted rather as an guiding example at low angles. Nonetheless, we can conclude that when the applied field exceeds the matching field, the pinning effiency is slightly decreased at intermediate angles because at low angles the pinning is generally strong and at very high angles the splayed vortices can get more easily pinned into several pinning sites thus increasing the total pinning force and *J*_*c*_.

For 12 nm diameter nanorods, presented in Fig. 2c, the *c*-peak starts to form only at higher field near *B*_*ϕ*_, which is opposite to the previous cases. This is due to the low number of wide BSO nanorods. At low field, where there are less vortices, the probability of the vortex coming across a nanorod is highly increased as the angle *θ* increases. In the low field range, the vortices can also bend and pin simultaneously into several nanorods. As the field and the number of vortices are increased, the repulsive vortex-vortex interactions prevent single vortices getting pinned simultaneously into several pinning sites, thus reducing the *J*_*c*_ at high angles. At low angles, the vortex-vortex interaction increases the probabilities of the vortices getting pinned and also in many cases the vortices were found stable outside the nanorods due to the repulsive interaction of nearby pinned vortices. These observations explain the shapes of the simulated *J*_*c*_(*θ*) curves in Fig. 2c.

### Effect of nanorod diameter

**Figure 3:**
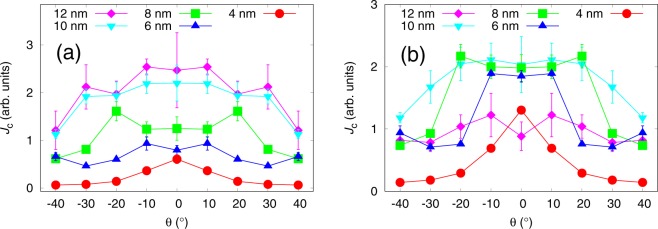
Simulated *J*_*c*_(*θ*) curves with standard errors for lattices of (**a**) constant number of 20 rods with varying diameters between 4–12 nm, respectively, and (**b**) nanorods of varying diameter and varying number corresponding to 4% consentration. The absolute values of *J*_*c*_ curves are comparable between (**a**,**b**). Zero angle is along the YBCO *c*-axis.

**Figure 4:**
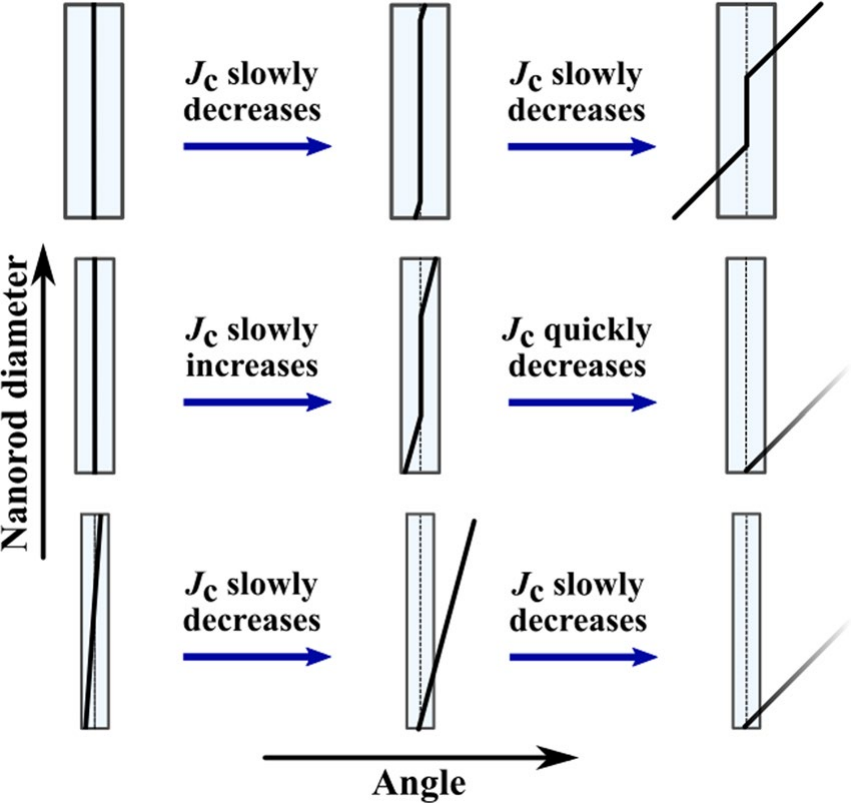
Schematic diagrams illustrating vortex pinning as a function of nanorod diameter and angle of applied field.

 Figure 3a presents the simulated *J*_*c*_(*θ*) curves for pinning site configurations of the same number of 20 nanorods but with a varying diameter. All the simulations were run below *B*_*ϕ*_ with 14 vortices. The absolute value of critical current increases somewhat directly proportionally to nanorod diameter with nanorod diameters up to 10 nm above which increasing nanorod diameter starts to have less impact on *J*_*c*_. The shape of the *c*-axis peak also changes dramatically with increasing nanorod diameter. The smallest 4 nm diameter nanorods produce sharp low-intensity *c*-peak. This is due to the very small pinning force of the 4 nm nanorods which is much smaller compared to the magnetic force that is aligning the vortex along the applied magnetic field. Thus at higher angles, the vortices can get only partially pinned to the nanorods resulting in decreased pinning force. The *J*_*c*_ increases at lower angles because vortices are more aligned with the nanorods resulting in increased pinning force. This has been schematically illustrated in Fig. 4. Weakness of 4 nm diameter nanorods is also manifested at high angles, where some vortices were observed to get trapped outside the nanorods due to vortex-vortex interactions.

In the case of nanorod diameters above 6 nm, the pinning force is increased so that its magnitude exceeds the magnetic force and pinning centers can align the vortices along them more efficiently as the nanorod diameter increases, as illustrated in Fig. 4. This explains the broadening of the peaks as nanorod diameter increases. The double peak structures observed for 6 nm and 8 nm nanorod diameters are found at the highest angles at which the nanorods are able to align the vortices along them. The highest *J*_*c*_(*θ*) is observed at this angle because the vortices are aligned optimally so that the product of their pinning force and probability to get pinned due to the splayed figure is maximized. Above this angle, the vortices get only partially trapped which dramatically decreases the total pinning force and thus *J*_*c*_, as for the 4 nm diameter case.

The 10 nm and 12 nm diameter nanorods are so strong that they can gather vortices to themselves at a distance thus resulting in maximum *J*_*c*_(*θ*) at zero angle. For 12 nm nanorods even multivortex trapping was observed. When the angle of applied magnetic field is increased, the magnetic force only barely bends the pinned vortices, as illustrated in Fig. 4, resulting in only a slight decrease in total pinning force and thus *J*_*c*_. At angles above 30^°^, the magnetic force overcomes the pinning force resulting in partially trapped vortices and decreased total pinning force. Fig. 4 summarizes the above discussion of how nanorod diameter affects the *J*_*c*_ at certain angles. Fig. 3b

presents the simulated *J*_*c*_(*θ*) curves for pinning site configurations where the number of nanorods have been varied so that their number would correspond to 4% concentration. As before, all the simulations were run below matching field with 14 vortices. Unlike in the previous case, now the ratio between applied field and matching field *B*_*ϕ*_ changes, which dramatically affects both the absolute values and shapes of *J*_*c*_(*θ*) curves. The two competing processes in the formation of *J*_*c*_(*θ*) curves are i) increasing nanorod diameter, that increases the absolute value of *J*_*c*_ as well as broadens the *c*-axis peak as observed above and ii) the decreasing *B*_*ϕ*_ as nanorod diameter increases, which decreases the absolute value of *J*_*c*_ and narrows the *c*-axis peak as observed in the previous section. From Fig. 3b we see, that the absolute value of critical current increases and the *c*-peak clearly broadens with increasing nanorod diameter between 4 nm and 6 nm. For nanorod diameters >6 nm, the effects of the matching field start to take place as the absolute values of *J*_*c*_ start to decrease and the *J*_*c*_(*θ*) curves get more isotropic as the nanorod diameter increases.

## Effect of Nanorod Concentration

**Figure 5:**
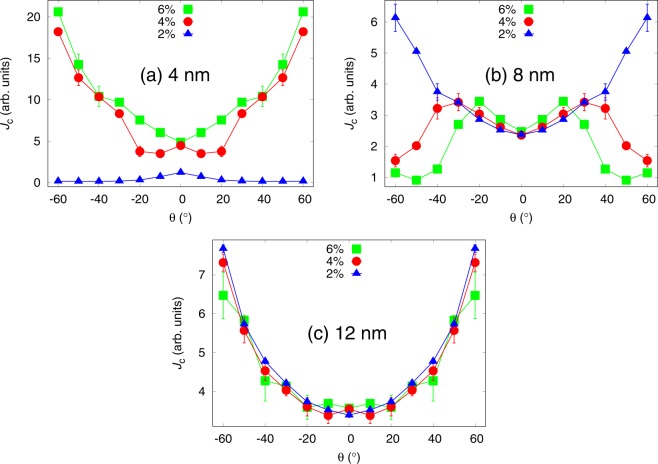
Simulated *J*_*c*_(*θ*) curves with standard errors for lattices of 4 nm, 8 nm and 12 nm diameter nanorods with concentrations of 2%, 4% and 6%. The number of vortices in each case is changed so, that the ratio between them and the number of nanorods is 0.2. The absolute values of *J*_*c*_ curves are comparable between each of the cases. Zero angle is along the YBCO *c*-axis.

Figure 5 presents the simulated *J*_*c*_(*θ*) curves for 2%, 4% and 6% concentrations for each 2 nm, 4 nm and 6 nm diameter nanorods, respectively. In order to eliminate the effects of matching fields discussed before, the simulations were run in different applied magnetic fields so that the ratio between vortices and nanorods was 0.2.

**Figure 6:**
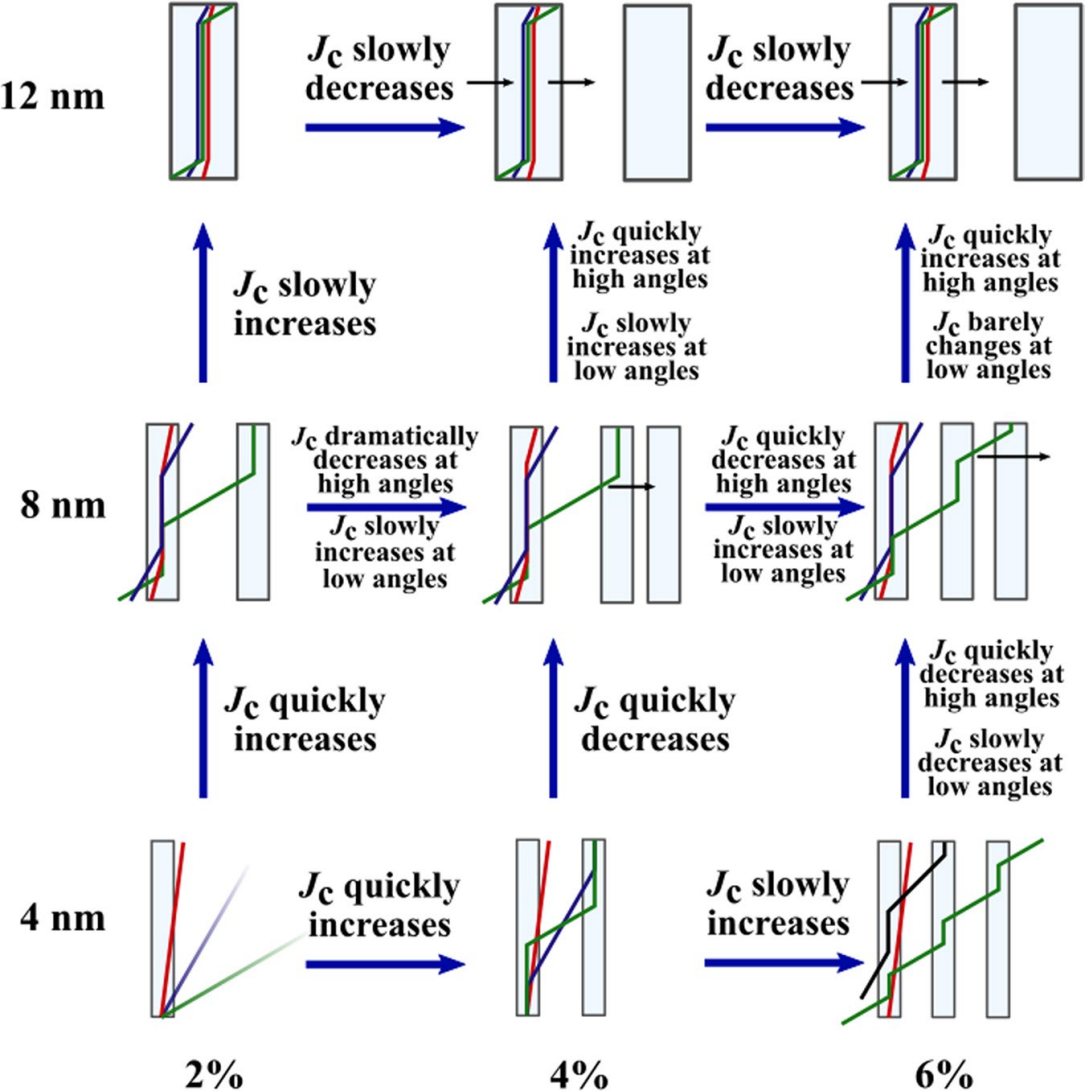
Schematic diagram of vortex pinning mechanisms in different diameter nanorods and consentrations. Different line colors represent vortices at different angles of the applied field. Presence of the NOD effect is indicated with black arrows.

Figure 5a presents the simulated *J*_*c*_(*θ*) curves for 4 nm diameter nanorods of different concentrations. For 2% concentration, a sharp low-intensity *c*-peak is observed. This can be explained with the same effect already described in section 3, where it was concluded that weak pinning sites that cannot overcome the magnetic force and align the vortices along them, thus resulting in maximum pinning force at zero angle. For the 2% dopant concentration, the nanorods are yet so far away from each other that a single vortex cannot get simultaneously pinned into several pinning sites. For 4% and 6% concentrations, on the other hand, the amount of nanorods is increased so much that the vortices are able to trap simultaneously into several different nanorods thus increasing the total pinning force, and thus *J*_*c*_, dramatically at high angles. This is illustrated in  Fig. 6.

For the 8 nm diameter nanorods, the pinning force is higher but their number is dramatically reduced, which seems to play a crucial role in the *J*_*c*_(*θ*) curves presented in Fig. 5b. For 2% concentration no *c*-peak is observed. This is intuitively expected, since vortices are less likely to come across a pinning site due to small number of nanorods. Instead, the vortices get much more easily trapped at higher angles when they can more easily entangle to several different nanorods due to the increased pinning force of the nanorods that tend to lure another ends of partially pinned vortices in their direction, as illustrated in Fig. 6. For 4% and 6% concentrations, the number of nanorods is increased so that *c*-axis oriented pinning becomes dominant which is seen as a clear *c*-peak that clearly gets narrower at 6% concentration. For these concentrations, the nanorods are so tightly arranged that it surprisingly disturbs vortex pinning at angles over 20^°^ as nearby unoccupied nanorods lure partially pinned vortices towards them making it difficult for the vortex lattice to stabilize. This *nanorod overdoping effect* (NOD effect) is rather an interesting result since it suggests that increasing the number of pinning centers does not in fact increase the absolute value of *J*_*c*_ linearly, but rather has an optimal concentration above which the *J*_*c*_ starts to decrease. The reader should be notified that this effect is not connected to the experimentally observed effect of the critical current decrease due to reduction of superconducting volume in the films as a result of high dopant concentrations nor it is the well known flux creep^28^.

The NOD effect can be explained quantitatively by assuming that the pinning rate of the vortices is proportional to (i) total number of vortices *N*_v_, (ii) the average speed of vortices *v*_v_, (iii) consentration of nanorods *n* and (iv) probability of vortex getting pinned ${p}_{{\rm{p}}} \sim (1-\Theta )\exp (-{E}_{{\rm{p}}}/kT)$, where Θ is percent of pinning centers that occupy a vortex and *E*_p_ is the energy difference between free and pinned vortex. The depinning rate of vortices, on the other hand, is proportional to (i) current dencity *J* and (ii) probability of vortex getting depinned ${p}_{{\rm{dp}}} \sim J\Theta \exp (-{E}_{{\rm{dp}}}/kT)$, where *E*_dp_ is the energy that vortex needs to get depinned. At equilibrium state, the pinning and unpinning rates are equal. With the assumption that the critical current is directly proportional to the percent of pinned vortices^29^ this leads to relation

1 $${J}_{c}(n) \sim \frac{{n}^{2}}{Bn+\exp (\Delta E/kT)},$$

where Δ*E* = *E*_p_ − *E*_dp_ > 0. Detailed derivation of Eq. 1 is presented in SI. Intuitively, since at least at high angles the vortices can get pinned into several nanorods simultaneously, Δ*E* has to be a function of nanorod concentration, so it can be postulated that $\Delta E \sim {E}^{{\prime} }{n}^{x}$ where *x* ∈ IR^+^. Our numerical study then implies that, under very reasonable assumption that the work done by the Lorentz force ${E}^{{\prime} }\gg kT$, function *J*_*c*_(*n*) has a maximum inside the range *n* ∈ [1, 0] regardless of parameters *B* and *x* ∈ IR^+^. The fact that *J*_*c*_ reaches a maximum after which it starts to decrease explains the observed anomaly in the simulations, where increasing the concentration of 8 nm and 12 nm diameter nanorods counterintuitively decreases the absolute value of *J*_*c*_.

For 12 nm nanorods the number of nanorods is even more decreased which results in decreased *c*-axis oriented pinning as seen from Fig. 5c similarly to 8 nm nanorods 2% case. In the case 12 nm nanorods though, the amount of pinning sites is so low that single vortex can only get trapped in a single nanorod. The value of *J*_*c*_ increases at high angles because of the enhanced probability of the vortex to meet a pinning site and get pinned. The absolute value of *J*_*c*_ surprisingly decreases as a function of concentration which is a result of the NOD effect presented before. In this case the pinning forces of nanorods are so strong that as vortex arrives with high speed to initial nanorod, a second nanorod behind it attracts the vortex and immediately unpins it from the initial nanorod etc. Figure 6 summarizes how increasing of nanorod diameter or concentration affects the *J*_*c*_ at certain angles.

### Comparing simulations to experimental data

#### General factors behind discrepancies

A huge factor behind the discrepancies between simulated and experimental data is modelling the nanorods as solid and perfectly *c*-axis oriented entities, which definitely is not the case in reality where the nanorods can be fragmented and splayed and even their diameter may vary substantially^13^. This might have surprising effects on the *J*_*c*_(*θ*) especially as the nanorod concentration is increased.

Other discrepancy factors, that are not directly related to the properties of the nanorods, include the natural pinning sites, such as threading dislocations and stacking faults, which in reality, unlike in our simulation, always occur in the films along with the nanorods^30^. These natural pinning sites have an effect on the matching field, which already by itself is somewhat ill-defined outside the idealized simulation model. Also, the precence of nanorods creates additional natural pinning sites, namely misfit dislocations, oriented parallel to to the nanorods around them^4^. The diameter around these natural pinning sites, where the superconductivity is suppressed, is much smaller than the YBCO coherence length and thus the superconducting order parameter cannot vary substantially in the vicinity of such natural pinning sites resulting in very small pinning forces^30^. Since the pinning forces created by the natural pinning sites can be concidered to be negligible when compared with the actual nanorods, along with computational reasons, the effects of the natural pinning sites have not been taken into account in the simulations. In addition to this, microstrain induced oxygen deficiencies around the nanorods increase their effective pinning radius. The oxygen deficiencies can occur over a radius of a few nanometers from the nanorods and their effect on the critical current varies over temperature^31,32^. The effect of this on the pinning potential, and thus force, is elusive to estimate, and thus it has not been explicitly taken into account in our simulation model.

In addition, while the experimental data presented in the previous subsections is measured at 40 K, our simulation does not take thermal effects into account. In some cases this might be a reason behind discrepancies between experimental and simulated data. Still, in this comparison of simulated and experimental data, the 40 K data shows good resemblance with the simulations. This might be due to the fact that the thermal effects in most of the cases are very minor compared to the effect of other varied quantities or, perhaps, because of other pinning centers in the YBCO lattice, that are not present in the idealized simulation model, somehow deflate the thermal escape of pinned vortices.

Finally, the degradation of superconducting properties due to doping cannot be included in the simulation, thus hindering the direct comparison of the absolute values of *J*_*c*_ between measured and simulated data.

#### Effect of applied magnetic field

**Figure 7:**
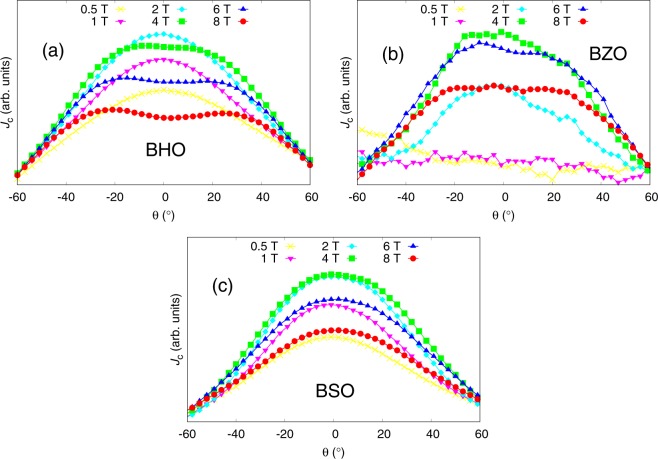
Experimentally measured *c*-axis peaks in varying magnetic fields for 4% BHO, BZO and BSO doped samples at 40 K.

Experimentally measured *J*_*c*_(*θ*) curves around YBCO *c*-axis in various magnetic fields for 4% BHO, BZO and BSO doped YBCO are presented in Fig. 7. Although, the real value of the matching field is extremely difficult to estimate in reality, some similarities can be seen between these and the simulated *c*-peaks of the different diameter nanorods, somewhat corresponding to those of BHO, BZO and BSO, in varying matching fields in Fig. 2.

In the simulations run for 4 nm diameter nanorods presented in Fig. 2a, one can see some similarities with the experimentally measured *c*-peaks for BHO in Fig. 7a. Generally, the simulated data resembles the experimental one by having a clear *c*-axis peak in all of the simulated fields above 0.1 ⋅ *B*_*ϕ*_. At high fields, we observe both experimentally and computationally the decrease in the peak intensity as the field increases although the sharpening of the *c*-peak cannot be observed experimentally. In this case, the differences between experimental and simulated data are minor when one takes into account the idealized simulation model where no nanorod splay, fragmentation or other type of defects are present.

The simulated *c*-peaks for 8 nm diameter nanorods, presented in Fig. 2b, correspond surprisingly well to the experimentally measured peaks presented in Fig. 7b. Firstly, at 0.5 T field, where we can assume being well below the matching field, one can observe a small increase in *J*_*c*_ around 40^°^ without any *c*-peak, which was also observed in the simulated curve at 0.25 ⋅ *B*_*ϕ*_. When the field is increased up to 4 T, the *c*-peak emerges and gets more pronounced until the *J*_*c*_ starts to decrease with flattened *J*_*c*_(*θ*) curve at 6 T and 8 T. In this field region, we are presumably getting closer to the matching field, without exceeding it, which would be in line with the simulated curves in Fig. 2b, where one can observe both decreasing and flattening of the *c*-peak as matching field is approached.

In the case of 12 nm diameter nanorods, a small *c*-peak starts to rise only very near to the matching field as seen from Fig. 2c, while experimentally we observe, from Fig. 7c, the high intensity and broad *c*-peak even at low field range. We argue that the discrepancy between experimental and simulated data is due to differences in the nanorod densities between reality and the simulated model a well as the effect of thermal escape of the vortices, which should be more probable at high angles where the vortices are only partially pinned. The effect of density is further discussed in section 5.4 in the case of BSO nanorods.

#### Effect of nanorod diameter

**Figure 8:**
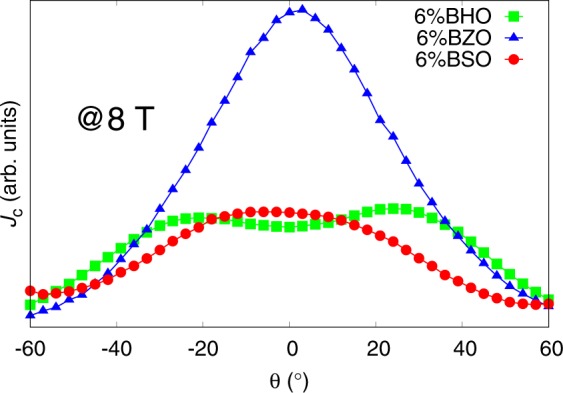
Experimentally measured *c*-axis peaks for 6% BHO (4 nm), BZO (7 nm) and BSO (12 nm) doped YBCO.

Experimentally measured *J*_*c*_(*θ*)-curves around YBCO *c*-axis at 8 T, which we presume to be well below the matching field, for 6% BHO (4 nm), BZO (7 nm) and BSO (12 nm) doped YBCO are presented in Fig. 8. We want to point out that the doping percent we are referring here is the doping concentration of the PLD target, which might differ from the doping concentration of the deposited film. Despite this, clear similarities can be seen between experimentally measured peaks in Fig. 8 and simulated *c*-peaks in Fig. 3b. Firstly, small diameter BHO nanorods produce a sharp but low intensity *c*-peak as observed in the simulations. Intermediate diameter BZO nanorods on the other hand produce sharp and pronounced *c*-peak, also clearly seen from the simulated curves in Fig. 3b. Finally, for large diameter BSO nanorods we observe a low intensity flattening *c*-peak, with slight double peak structure, exactly what was observed in the simulations.

#### Effect of nanorod concentration

**Figure 9:**
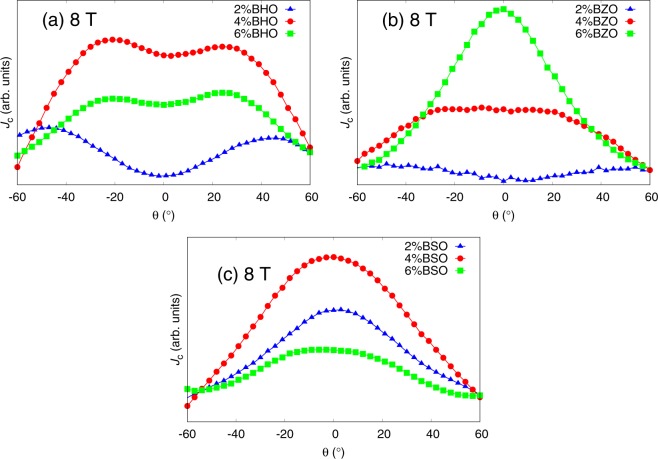
Experimentally measured *c*-axis peaks for 2wt%, 4wt% and 6wt% BZO doped YBCO.

For all of the reasons listed at the beginning of this section, the pure effect of concentration on the shape of the *c*-peak is extremely difficult to compare with experimental data, although some similarities can be seen between the experimentally measured *c*-peaks of the BZO nanorods and the 4 nm diameter nanorod simulations, presented in Figs. 5b and 9b, respectively. In the case of BHO and BSO nanorods, we believe that, especially since these nanorods represent the extremes of the diameter range, the discrepancies between experimental observations and simulations are related to much greater density of nanorods in the film when compared to our simulation model. For BHO, this agrees with the matching field simulations presented in Fig. 2a, assuming that even at 8 T field the matching field is not exceeded at least for 2% and 4% doped samples. For BSO, the highest *c*-peak is observed experimentally for 4% doped sample, as seen in Fig. 9c, indicating that at 8 T the matching field might have been exceeded between 4% and 6% dopant concentrations. This observation is in line with the fact that the concentration of the nanorods is greater in reality than in the simulation which is at least one of the the main reasons behind the observed discrepancies between experimental and simulated data. A simulation with 8% concentration of 12 nm diameter nanorods at 0.5 ⋅ *B*_*ϕ*_ was also run in order to support the above explanation. The simulation indeed reveals a clear *c*-peak as we had speculated, which provides a quantitative proof for the density differences between real and simulated nanorod lattices. The thermal effects, for sure, also play a role in this, especially since it is a question about the effect of concentration, although it is hard to estimate this effect is in reality.

### Flux pinning mechanisms in BHO, BZO and BSO doped YBCO films

**Figure 10:**
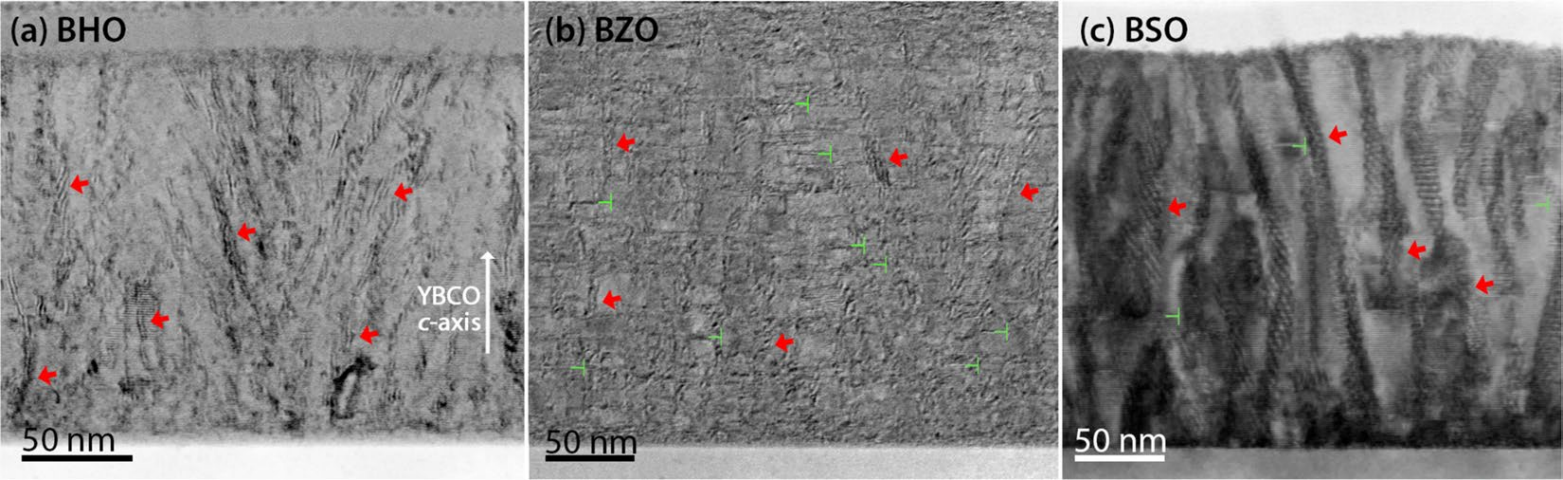
Cross-sectional BF-STEM view images of 4% (**a**) BHO, (**b**) BZO and (**c**) BSO doped YBCO films. Nanorods and line defects are marked with arrows and mark tones, respectively.

In order to study the flux pinning mechanisms in BHO, BZO and BSO doped YBCO films their microstructure was investigated with birght-field scanning transmission electron microscopy (BF-STEM). The parameters, such as nanorod diameter, splay, fragmentation etc., obtained from the results were then used to create pinning site configurations for the simulations that mimic the real life situation as precisely as possible. The experimental data used in this section was measured at 10 K, since at this temperature the interesting rise of the *c*-peak at 1 T was observed, the underlaying mechanism of which we wanted to study. The cross-sectional BF-STEM images of 4% BHO, BZO and BSO doped YBCO films are shown in Fig. 10 and the parameters obtained from BF-STEM analysis are presented in Table 2. The high-resolution cross-sectional BF-STEM images are presented in SI.

**Table 2:** The average diameter, tilting angle measured from YBCO *c*-axis (splay) and length of different nanorods obtained from BF-STEM measurements along with the associated sigma values.

Nanorod	Diameter (nm)	Splay (^°^)	Length (nm)
BHO	4.0 ± 0.7	11.8 ± 0.4	44.7 ± 7.9
BZO	6.8 ± 2.3	10.9 ± 0.3	29.1 ± 8.7
BSO	11.9 ± 3.2	8.8 ± 0.6	111.4 ± 17.6

The simulations were run for all the nanorods, but only the simulations for BZO and BSO produced results that were comparable to experimental data. In the case of BHO, where the nanorod diameter is 4 nm at its largest, the simulated *J*_*c*_(*θ*) curves produce sharp *c*-axis peak that flattens quickly as angle increases. Experimentally we see quite the opposite, as no *c*-peak is observed and *J*_*c*_ increases as the angle is increased. This can be explained by the fact that in reality, there exists a lot of other type of defects that act as pinning centers which can significantly increase and broaden the *ab*-peak. This in turn, can easily disturb the low intensity *c*-peak observed in the simulations so much that it is experimentally invisible.

The simulation parameters for BZO and BSO are presented in Table 3. Nanorod positions and the orientations of splay in a single layer were randomly generated. The number of nanorods was calculated to match 4% concentration so that the ratio of total cross-sectional area of nanorods and area of superconducting material equals 0.04. The fragmentation of the nanorods was neglected because, although the BF-STEM measurements reveal that the nanorods are fragmented, the nanorod fragments are aligned next to each other so that they form, as a whole, somewhat continuous columnar pinning site from the bottom to the surface of the film, as seen from Fig. 10. This kind of strong deformation of YBCO between the BZO particles and their formation as a continuous columnar pinning site is also expressed earlier in^33^. The number of vortices used in every simulation corresponds to 0.5 T, and 1 T field which is well below *B*_*ϕ*_ for BZO but slightly above *B*_*ϕ*_ for BSO in 1 T. These fields were chosen because the availability of experimental data and the fact, that the simulation does not perform well too much above *B*_*ϕ*_. In reality, the matching field is an ill-defined concept which is extremely hard, if even possible, to estimate from the two-dimensional BF-TEM images. According to the analysis done in section 2, very roughly speaking, one can state that for BZO doped sample, at fields 0.4 ⋅ *B*_*ϕ*_ < *B* < 0.8 ⋅ *B*_*ϕ*_, the general shape of the *c*-peak does not change. For large BSO nanorods this field range is even wider. Since the simulated *J*_*c*_(*θ*)-points have to be scaled to fit the experimental data and the field range is believed to be so, that the shape of the *c*-peak is not much affected, no further effort has been put to evaluating the matching fields in reality.

**Table 3:** The parameters used in pinning site configurations for simulations involving different nanorods. These include nanorod diameter *d*, tilting angle of the nanorods measured from YBCO *c*-axis (splay) Φ, number of nanorods in a single layer *N*_R_, and number of vortices *N*_V_ corresponding to 0.5 T and 1.0 T fields, respectively.

Nanorod	d (nm)	*Φ* (^°^)	*N*_R_	*N*_V_ (at 0.5 T)	*N*_V_ (at 1 T)
BZO	3.4	11	42	9	19
BSO	6.0	9	14	9	19

**Figure 11:**
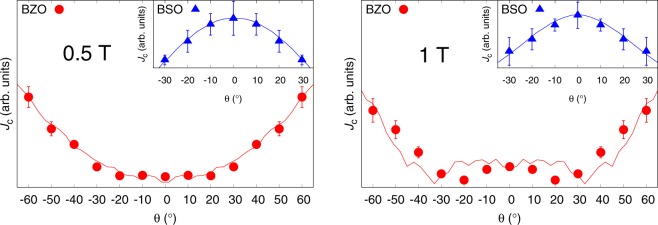
Simulated data points of 4% BZO and BSO doped YBCO films presented with experimentally measured *J*_*c*_(*θ*) curves (solid lines) in 0.5 T and 1 T applied fields. The simulation corresponds to the pinning structure observed by BF-STEM.

The simulated *J*_*c*_(*θ*) points and their corresponding experimental data are presented in Fig. 11 for 0.5 T and 1 T fields, respectively. The simulations for BZO resemble the shape of the experimentally measured *J*_*c*_(*θ*) curve with great precision for all the simulated angles despite very rough and simple modeling of the pinscape. For BSO, the simulation fails at angles above  ±30^°^ due to exceeding of the matching field, since the vortices are hard to get stabilized outside the nanorods.

The simulations reveal, that the pinning efficiency to BZO nanorods is weakest at intermediate angles 30^°^ - 40^°^ because at this angle the vortex is splayed enough so that the nanorods are not able to align them along their direction, but also not splayed enough that the vortices could get pinned into several nanorods simulataneously. The rising of the *c*-peak when field is increased from 0.5 T to 1 T, which are both below *B*_*ϕ*_, is due to increased vortex-vortex interaction that improves pinning efficiency at low angles as the pinned vortices block the movement of free vortices. At higher angles, the significance of this effect is reduced since the vortices can effectively trap into several nanorods simultaneously. At angles *θ* < 10^°^ the *J*_*c*_ stays roughly constant because the nanorods are able to align the vortices almost completely along their direction at these angles. Although these results are not exactly comparable to analysis done in the section 2 due to different nanorod size and density, one can still see many similarities between these cases, for example the disappearance of *c*-peak at low field.

In the case of BSO nanorods, the observed high intensity *c*-peaks are due to very strong BSO nanorods that can gather vortices to themselves at a high distance and align them along their direction. As the angle increases this alignment becomes less effective and due to small number of nanorods vortices cannot get pinned into several nanorods simultaneously, reducing the pinning force and thus *J*_*c*_ as the angle increases. This results in smooth *c*-peak, as concluded in section 3.

## Conclusions

We have extensively investigated the effects of the applied magnetic field, nanorod size and their concentration on the critical current isotropy and explained them via the dynamics of the vortices. In summary, we have shown and explained how the shape and height of the *c*-axis peak is highly dependent on the ratio between applied and matching fields and how increasing the nanorod diameter and concentration affect the formation of the *c*-peak. We have comprehensively compared and discussed the similarities and discrepancies between the experimental and the simulated data. The experimental *J*_*c*_(*θ*) data was also directly compared to the simulations based on BF-STEM measurements with with good success for BZO and BSO nanorods.

We have also observed and quantitatively explained a very unintuitive result referred as nanorod overdoping effect, which states that even in the ideal case, in which we disregard the decreasing of superconducting properties as dopant concentration is increased, increasing the dopant concentration, ultimately, starts to decrease the critical current after some optimal concentration due to nearby nanorods luring the partially pinned vortices towards them.

## Methods

### Thin films fabrication

4% BHO, BZO and BSO doped YBCO films were grown on SrTiO_3_ (STO) (100) substrates using the pulsed laser deposition method (PLD). Target materials, manufactured by solid state ceramic method as described in^26,34^, were irradiated with 308 nm XeCl excimer laser using 1600 pulses with 5 Hz deposition rate and 1.3 Jcm^−2^ fluence. The depositions were done at 750 ^°^C with 0.175 Torr oxygen flow after which temperature was decreased down to 725 ^°^C for 10 min oxygen treatment at 750 Torr. Further details of the deposition process are given in^26,35^.

### TEM measurements

Nanorod size and orientation were investigated with JEOL JEM 2200FS bright-field scanning transmission electron microscope using 200 kV operating voltage. For this purpose, cross-sectional lamellas of the samples were cut using Focused Ion Beam technique in a FEI Nova 600 Nanolab Dual Beam FIB-SEM and extracted by Omniprobe extraction needle using *in situ* lift out procedure^36^. The image processing software *ImageJ* with statistical measurement was used to calculate the average values of nanorod diameter, splay and size in several cross-sectional areas (high and low resolution images).

### Transport measurements

To investigate the anisotopy of angular dependent critical current *J*_*c*_(*θ*), all the films were first patterned by wet chemical etching in order to produce 50 *μ*m wide stripes. Electrical contacts were made with aluminium wire using TPT HB05 Wire Bonder. The transport measurements were done by four-point contact method with Quantum Design PPMS system using the horizontal rotator option in the field and temperature ranges of 0.5 T–8 T and 10 K–77 K, respectively. The angular range was 0^°^–360^°^ with 3^°^ steps.

### MD simulations

A critical current angular dependency MD simulation, which is described comprehensively in^25^, was used to study the effects of applied field and the dimensions and concentration of the nanorods as well as to make quantitative connections between experimentally measured transport properties and nanorod microstructure in YBCO thin films. Details of the simulation model are also given in Supplementary information (SI).

## Supplementary information

Supplementary Information.
