# Supplementary material for: Self-assembled nanorods in YBCO matrix – a computational study of their effects on critical current anisotropy
Source: Sci Rep. 2020 Feb 21;10:3169. doi: 10.1038/s41598-020-59879-3 (PMC7035360; doi:10.1038/s41598-020-59879-3)
Supplement: Supplementary file 1 — Supplementary Information. [file 41598_2020_59879_MOESM1_ESM.pdf]

Supplementary Information: Artificial pinning centers in YBCO matrix - the effect of nanorod size, concentration and applied magnetic field on critical current anisotropy

Elmeri Rivasto[1,2,*], Mukarram Zaman Khan[1,2], Mika Malmivirta[1], Hannes Rijckaert[3], Moe Moe Aye[1], Teemu Hynninen[1], Hannu Huhtinen[1], Isabel Van Driessche[3], and Petriina Paturi[1]

[1]Wihuri Physical Laboratory, Department of Physics and Astronomy, University of Turku, FI-20014 Turku, Finland
[2]University of Turku Graduate School (UTUGS), University of Turku, FI-20014 Turku, Finland.
[3]SCRiPTS, Department of Chemistry, Ghent University, Krijgslaan 281 S3, 9000 Ghent, Belgium
[*]elmeri.o.rivasto@utu.fi

1 Simulation model

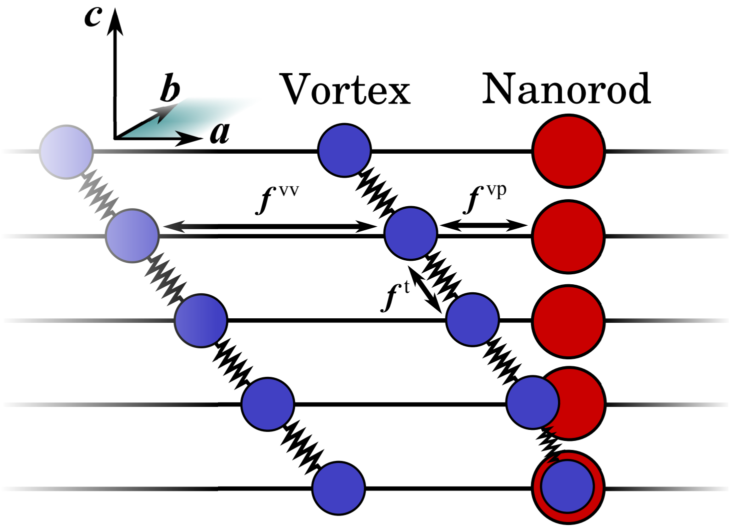

Figure S1. The schematic diagram of the simulation model with the most relevant interaction forces, namely the repulsive vortex-vortex interaction f^{vv}, vortex line tension f^{t} and attractive pinning force f^{p} between vortex and nanorod particles. Orientation of the lattice of YBCO is presented in the figure.

A detailed description of the molecular dynamics simulation model and its validity has already been given in[?], thus here we summarize only the most important features of the simulation model that are the most relevant regarding this article.

The simulation model is based on layer structure schematically illustrated in Fig. S1, in which vortices are modeled as chains of particles connected to each other via spring-like line tension force f^{t} that acts between two vortex particles of the same vortex located in adjacent layers. The vortex is also affected by the magnetic force f^{m} that strives to align the individual vortex particles along its angle θ. The vortex particles are also affected by Lorentz force that strives to keep the vortices moving and drag force that slows the movement of the vortices. The attractive pinning force acts between a vortex particle and a nanorod particle that are located in the same layers while the vortex-vortex interaction repels the vortex particles of the same

Table S1. Forces in the simulation. Parameters in the equations are vortex characteristic energy $\varepsilon_0 = \Phi_0^2/(2\pi\mu_0\lambda^2) \approx 2.76 \cdot 10^11$ J·m^{-1}, where Φ_0 in the magnetic flux quantum, penetration depth and coherence length along the YBCO *ab*-plane $\lambda_{ab} = 140$ nm and $\xi_{ab} = 1.5$ nm, respectively, the magnetic permeability of vacuum μ_0, applied magnetic field B, normal state resistivity of YBCO $\rho_n \approx 5.3 \cdot 10^{-7}$ Ωm, the upper critical field of YBCO in the *c*-direction at 77 K $B_{c2} \approx 27$ T, applied current density $\mathbf{J}$ and the direction of the vortex $\mathbf{e}_V$, angle dependent Blatter scaling parameter $\varepsilon = (\sin^2(\theta)/\gamma^2 + \cos^2(\theta))$, where $\gamma \approx 5.0$ is the anisotropy parameter of YBCO and θ is the angle measured from YBCO *c*-axis, d is the distance between adjacent vortex particles in *c*-direction and r is their distance in the *ab*-plane. The only force that acts between adjacent layers is the vortex line tension.

Name of the force	Equation
Vortex line tension	$f^t = -\dfrac{\varepsilon_0 r(\gamma^2 - 1 + \ln\kappa)}{d\gamma^2\sqrt{d^2+r^2}}$
Magnetic force	$f^m = \Phi_0\mu_0 B\sin(\theta)\dfrac{d\theta}{dr}$
Drag force	$\mathbf{f}^d = -\dfrac{\Phi_0 B_{n2}}{\rho_n}\mathbf{v}$
Lorentz force	$\mathbf{f}^L = \mathbf{J} \times \Phi_0\mathbf{e}_V$
Vortex-vortex interaction	$f^{vv} = \dfrac{\varepsilon_0}{\lambda_{ab}}\mathrm{K}_1\left(\dfrac{r}{\lambda_{ab}}\right)$
Pinning force	$f^{vp} = \varepsilon_0\dfrac{rr_0^2}{\left(r^2+2\varepsilon\xi_{ab^2}\right)^2}$

layer away from each another. The total force acting on vortex particle n in the layer i can thus be calculated from

$$\mathbf{F}_{i,n}^{\text{tot}} = \sum_{j=i\pm1}\mathbf{f}^{t}_{(i,n),(j,n)} + \sum_{j=i\pm1}\mathbf{f}^{m}_{(i,n),(j,n)} + \sum_{m\neq n}\mathbf{f}^{vv}_{(i,n),(i,m)} + \sum_{k}\mathbf{f}^{vp}_{(i,n),(i,k)} + \mathbf{f}^{d}_{(i,n)} + \mathbf{L}^{d}_{(i,n)}. \tag{1}$$

The value of the critical current at certain angle was obtained iteratively by using the bisection method where the absolute value of the current was adjusted until a certain stability of the vortex system was achieved. In order to get good statistics, for a certain simulation five different pinning site configurations were randomly generated for which $J_c(\theta)$s were calculated separately. The simulations were run in a grid equivalent to $4 \cdot 10^4$ nm^2 or, as in some of the cases, $1 \cdot 10^4$ nm^2 size with periodic boundary conditions. The final $J_c(\theta)$ was then calculated as the average value, with standard errors, of the previously mentioned simulations.

Due to the layer structure of the simulation model, $J_c(\theta)$ curves can only be simulated up to $\pm 60°$ since above this angle the layer structure itself induces fictitious forces striving to align the vortex along the YBCO *c*-axis. Also, at high angles, the repulsion forces between the vortex particles located in different layers should be taken into account which is not the case in this simulation model.

2 Nanorod overdoping effect

The potential energy of the vortex in a pinning site can be approximated by a potential described in Fig. S2. We have defined pinning potential E_p and depinning potential E_{dp}, the difference of which, ΔE, is equal to the work done by the Lorentz force resulting from the transport current. We postulate that the pinning rate R_p of the vortices is proportional to the total number of vortices N_V, the average speed of the vortices v_v, number of nanorods, which is proportional to the dopant concentration n and the pinning probability p_p, so

$$R_p \sim N_V \cdot v_v \cdot n \cdot p_p. \tag{2}$$

The number of vortices is proportional to the applied magnetic field $N_V \sim B$ while the speed of the vortices is proportional to the transport current $v_v \sim J$. Assuming that no multivortex pinning occurs, the pinning probability is proportional to the number of free pinning sites as well as the Boltzmann factor. In order to express this mathematically, we introduce the occupation number $\Theta \in [0,1]$, which expresses the percent of occupied nanorods. With the help of this quantity, one can write the pinning probability as $p_p \sim (1-\Theta)\exp(-E_p/kT)$. With these relations, Eq. (2) can be written as

$$R_p \sim BJn(1-\Theta)e^{-E_p/kT}. \tag{3}$$

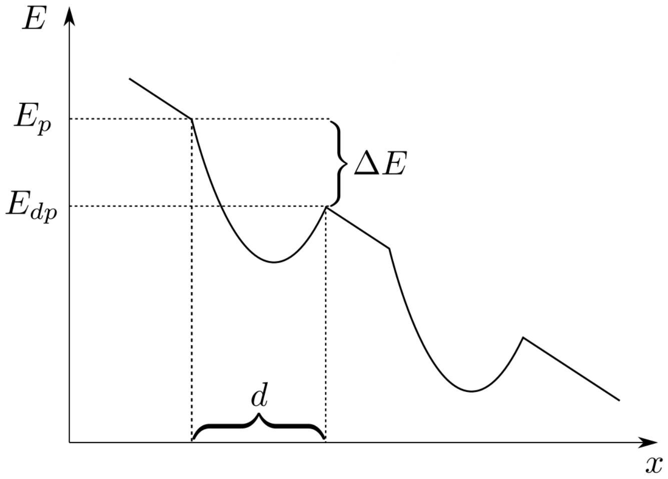

Figure S2. A schematic diagram of a vortex energy in a pinscape illustrating the pinning and depinning energies E_p and E_dp, respectively, and the diameter of the nanorod d. Position coordinate x is in a direction that is perpendicular to the transport current J.

The depinning rate of the vortices, on the other hand, is postulated to be proportional to the absolute value of the transport current J and depinning probability which is proportional to the number of pinned vortices and the Boltzmann factor, namely $p_\mathrm{dp} \sim \Theta \cdot \exp(-E_\mathrm{dp}/kT)$. Thus for the depinning rate, we get relation

$$R_\mathrm{dp} \sim J\Theta \mathrm{e}^{-E_\mathrm{dp}/kT}. \tag{4}$$

At equilibrium state $R_\mathrm{p} = R_\mathrm{dp}$. Thus, combining equations (3) and (4) and solving for Θ gives

$$\Theta = \frac{Bn\mathrm{e}^{-E_\mathrm{p}/kT}}{Bn\mathrm{e}^{-E_\mathrm{p}/kT} + \mathrm{e}^{-E_\mathrm{dp}/kT}}. \tag{5}$$

The critical current density is postulated being proportional to the percent of pinned vortices, namely $J_c \sim N_\mathrm{p}/B$. With the help of this, the occupation number can be written as

$$\Theta \sim \frac{N_\mathrm{p}}{n} \sim \frac{J_c B}{n}. \tag{6}$$

Inserting Eq. (6) into Eq. (5) and solving for J_c, we obtain

$$J_c(n) \sim \frac{n^2}{Bn + \mathrm{e}^{\Delta E/kT}}, \tag{7}$$

where $\Delta E = E_\mathrm{p} - E_\mathrm{dp}$ as illustrated in Fig. S2. Thus, we have obtained a relation between critical current density and nanorod dopant concentration. Intuitively, since at high angles vortices can get pinned into several nanorods simultaneously, we postulate that ΔE is a function of nanorod concentration of the form $\Delta E \sim E' n^x$, where E' and $x \in \mathbb{R}$. With this assumption, the maxima of $J_c(n)$, as given in Eq. (7), is elusive to solve analytically. Thus, the shape of $J_c(n)$ was investigated by plotting Eq. (7) inside range $n \in [0,1]$. The ratio E'/kT turns out to be the most critical parameter in finding the maximum of $J_c(\theta)$ at this range. The maximum is to be found regardless of parameters B and x, when $E' \gg kT$. This condition is easily fulfilled, since the work done by Lorentz force is in the range of

$$E' = J\Phi_0 d \sim 10^{-19} \quad \mathrm{J},$$

where d stands for nanorod diameter and thermal energy in superconductors is in the range of $\sim 10^{-22}$ J. This result implies that, even in the case of an ideal superconductor, increasing the number of nanorods in its lattice will eventually start to decrease the J_c after an optimal concentration is exceeded.

3 High resolution BF-STEM images

The high resolution cross-sectional BF-STEM images are presented in Fig. S3. These images, along with the standard low-resolution images presented in the main manuscript, were used to statistically calculate the average and sigma values of nanorod diameter and splay in several cross-sectional areas by *ImageJ*-program.

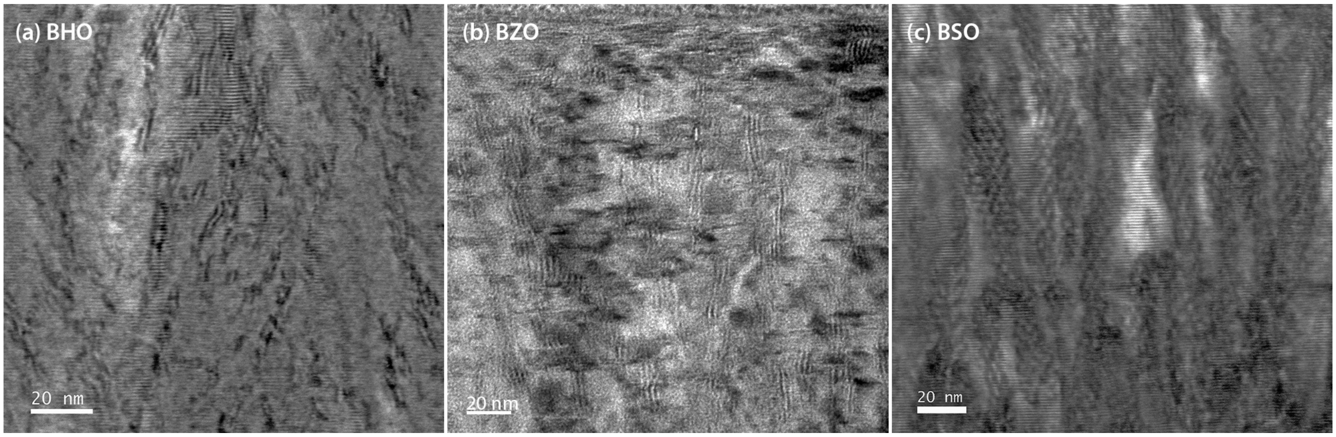

Figure S3. High-resolution cross-sectional BF-STEM view images of 4% (a) BHO, (b) BZO and (c) BSO doped YBCO films.
